# Developing Professional Identity Through a Student-Led Interprofessional Clinic: A Qualitative Study in Experiential Pharmacy Education

**DOI:** 10.1177/23821205261458512

**Published:** 2026-06-03

**Authors:** Ville Valkonen

**Affiliations:** 1 School of Pharmacy, 205537 University of Eastern Finland, Kuopio, Finland; 2 Hospital Pharmacy, Wellbeing Services County of North Savo, Kuopio, Finland

**Keywords:** pharmacy education, interprofessional education, student-led clinic, professional identity, curriculum development

## Abstract

**Objective:**

Structured models integrating pharmacy students into student-led interprofessional outpatient clinics remain limited in public healthcare contexts. This study evaluated an experiential pharmacy education model embedded within a newly established interprofessional health student clinic, examining its benefits and limitations for collaborative learning, role clarity, and professional identity formation.

**Methods:**

A six-week elective course was implemented within a newly established student-led interprofessional clinic in a primary care context in Finland. The model comprised structured orientation, supervised clinical practice including independent and joint interprofessional appointments, and guided reflection. A qualitative descriptive evaluation was conducted using thematic analysis of reflective diaries and written feedback of the pharmacy students; and instructor observations.

**Results:**

Nine fifth-year pharmacy students completed the course. Four central themes emerged: professional role clarification, increasing self-confidence through repeated patient encounters, the importance of structured orientation for clinical integration, and skill rehearsal and role integration in authentic clinical practice. The transition from independent pharmacist-led sessions to embedded interprofessional consultations was perceived as particularly influential in promoting collaborative role integration.

**Conclusion:**

Structured role integration within student-led clinics may strengthen professional identity formation and collaborative competence in pharmacy education.

## 1. Introduction

Interprofessional education (IPE) is widely regarded as essential for preparing healthcare professionals for collaborative practice, and it can improve aspects of professional practice.^1^ However, translating IPE from classroom-based activities into authentic clinical environments remains a global challenge. In today’s complex healthcare system, effective patient care depends on interprofessional collaboration, making systematic and well-designed IPE increasingly important.^2^ In this study, IPE is understood as structured learning in which students from two or more health or social care professions learn with, from, and about each other to strengthen collaboration, clarify professional roles, and support patient-centred care.^2^

Student-led interprofessional clinics have emerged internationally as a promising model for providing authentic, team-based experiential learning.^3-5^ Recent evidence suggests that such clinics benefit students, educators, and patients by supporting professional identity formation, role clarity, and collaborative competence. Clinical settings offer rich and dynamic learning environments for healthcare students, but the opportunities they provide are often implicit and not automatically realized.^6^ Simply placing students from different professions in the same space is insufficient; meaningful IPE requires structured experiential tasks with clear objectives, guidance, and assessment to fully realize the benefits of interprofessional collaboration.^7^ Experiential learning and student-led clinics are therefore increasingly viewed as foundational to professional identity formation.^8,9^

Work-based learning experiences within student-led clinics are valued highly among undergraduate pharmacy students.^10^ However, structured models integrating pharmacy students into student-led interprofessional outpatient clinics remain mostly limited in the public healthcare context. For example,in the United Kingdom student-led pharmacy clinics are still uncommon; current initiatives are largely emerging and exploratory rather than embedded within routine pharmacy education^11^ In contrast, experiential learning through structured service-learning is well-established in the United States, with more than 85% of pharmacy schools reporting its routine use.^12^

In Finland pharmacy education has traditionally included limited patient-care-oriented practical training in clinical setting.^13^ The Bachelor of Pharmacy curriculum requires two compulsory pharmacy placements, one of which can be completed in a hospital; however, both can also be completed in a community pharmacy.^14^ The recently introduced clinical pharmacy discipline for the master’s students at the University of Eastern Finland (UEF) is currently the only program within Finnish pharmacy education that includes mandatory clinical training alongside other work-life and patient-care oriented courses during the master’s level of study.^15,16^ In 2025, the Wellbeing Services County of North Savo launched a new interprofessional learning unit, Osmo.^17^ This unit provides practical, real-world learning opportunities for future health and social care professionals through student-led clinics in primary care context. The initiative was established in collaboration with the Wellbeing Services County of North Savo and local educational institutions, including UEF and Savonia University of Applied Sciences. During the starting phase, the unit trained and supervised students from diverse fields such as general medicine, nursing, dentistry, social sciences, physical therapy, nutrition science, dermatology, psychiatry, and pharmacy. Conceptually, the Osmo clinic can be understood as a Community of Practice (CoP).^18^

This study aimed to evaluate and analyze the benefits and limitations of a newly established student-led interprofessional teaching clinic through an experiential pharmacy education course. The study examined how the educational model supported the development of professional identity formation, clarified interprofessional roles, and highlighted the structural challenges of collaborative clinical learning by integrating pharmacy students into authentic patient care at the teaching clinic.

## 2. Methods

This exploratory study employed a qualitative descriptive design to evaluate the development of role clarity and professional identity among participating pharmacy students. A new elective course was established and conducted in autumn 2025 to operationalize and test the pharmacist’s role within a student-led interprofessional teaching clinic. The course comprised three structured phases: orientation, supervised clinical practice, and guided reflection, as outlined in Table 1.

**Table 1.** Key Stages and Activities in the Student-Led Interprofessional Clinic for Pharmacy Students

Stage	Pharmacy student activities	Instructor & teacher activities
Orientation	Role overview, system training, clinic workflow	Prepares orientation, provides guidance
Clinical practice	Independent medication reconciliation session, joint interprofessional appointment	Available for consultation
Reflection	Group discussion, learning diary, self-assessment, peer feedback	Facilitates reflection discussion, provides feedback, analysis of student feedback

The curriculum combined structured and experiential components. During orientation, students received guidance on clinical workflows, documentation practices, and the pharmacist’s role in patient care, supported by introductory sessions and practical system training. Training in the patient information system was not considered interprofessional learning in itself, but it supported participation in shared documentation and communication practices within the interprofessional clinic. The six-week educational program, held from September 1 to October 10, 2025, included weekly student-led patient appointments, in which various forms of pharmacy counseling were tested. During clinical practice, students conducted medication reconciliation and review in real patient appointments, either independently (in pharmacist-led sessions) or as part of interprofessional consultations. These encounters required students to prepare by reviewing patient records, conduct patient interviews, assess medication use, and communicate findings within the team.

The group of students attending the appointments varied from week to week. The pharmacy and nursing students attended every week during the six-week program, and students from the nutrition, physical therapy, oral health, and social work programs also attended in varying groups.

Appointments were student-led, with instructors present in a supervisory capacity. A nurse instructor attended all sessions to ensure patient safety and adherence to the protocols but intervened only when necessary. The pharmacy instructor participated only upon student request. Patients attending the student-led appointments were typically referred for nursing consultations as diabetes and arterial disease follow-ups, memory clinic visits or lifestyle counseling.

Learning was supported by supervision and daily facilitated reflection sessions after the appointments, where students discussed clinical decisions, role boundaries, and interprofessional collaboration. Reflection was guided using prompting questions focusing on professional roles, communication, and clinical reasoning. These sessions, facilitated by instructors of different disciplines on a rotating basis, included all available students and faculty.

A qualitative descriptive design was used to explore how pharmacy students experienced their professional role, identity development, and participation in interprofessional clinical learning during the six-week student-led clinic course. The qualitative dataset comprised reflective diaries, written course feedback, notes from facilitated interprofessional reflection discussions conducted at the end of clinic days, and instructor observations. These data sources were selected to capture both students’ individual reflections and shared reflections arising during supervised clinical practice. Data were analyzed using thematic analysis. The analysis began with repeated reading of all materials to develop familiarity with the dataset. Initial codes were generated from meaningful text segments describing students’ experiences of patient encounters, medication reconciliation, interprofessional appointments, documentation practices, role negotiation, and perceived learning needs. Codes were compared across data sources and grouped into broader categories reflecting recurring patterns in students’ experiences. Through iterative review and refinement, these categories were developed into themes describing role clarity, professional self-confidence and self-efficacy, enabling conditions for clinical integration, and skill rehearsal and role integration.

According to national guidelines, this project was classified as educational development and did not require formal ethical review. All students provided informed consent for anonymized use of their reflections. The reporting of this study conforms to Consolidated criteria for Reporting Qualitative research QOREQ) (Supplemental File 1).^19^

## 3. Results

**Figure 1.**
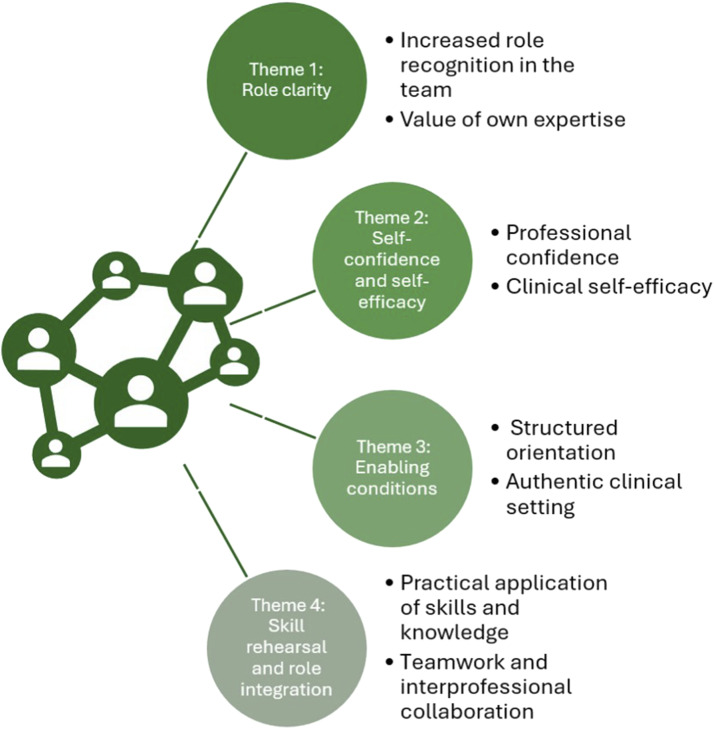
Key themes identified from qualitative analysis of pharmacy students’ reflections and feedback on interprofessional clinic experiences

Nine fifth-year pharmacy students participated in the course. Analysis of feedback and reflective diaries identified four central themes: (1) emergence of professional role clarity, (2) strengthening of professional self-confidence through repeated patient encounters, (3) the importance of structured orientation in supporting clinical integration, and (4) Skill rehearsal and role integration through practical traingin in authentic clinical setting (Figure 1). Throughout the course, pharmacy students participated in both independent pharmacist-led sessions and joint interprofessional appointments. Over time, joint consultations became the primary format, as they were perceived as more feasible and pedagogically effective.

### 3.1. Role Clarity

Participation in joint interprofessional appointments facilitated clearer articulation of the pharmacist’s role within team-based care. As students moved from independent sessions to integrated consultations, they increasingly described recognizing the scope and value of their expertise within the team. These perceptions emerged in the context of repeated, structured patient encounters combined with guided reflection, which allowed students to progressively test and refine their professional roles in practice.

*“These experiences have been invaluable in developing my professional identity and skills. I learned to better recognize the extent of my expertise and how to develop my teamwork skills.”*

*“I have gained practical knowledge about planning and managing a patient appointments. Seeing how roles are allocated, how the agenda is set, and how plans can be modified according to the situation has been useful. These experiences have been invaluable in developing my professional identity and skills”*

These reflections suggest a shift from task-oriented performance toward a more explicit professional identity within collaborative practice.

### 3.2. Self-Confidence and Self-Efficacy

Each pharmacy student had two to three patient appointments during the six-week course. Repeated patient encounters appeared to strengthen professional confidence. Early consultations were described as stressful due to unfamiliarity, whereas later appointments allowed students to focus more on clinical reasoning and their professional contribution.

*“The first appointments were a little nerve-wracking because everything was new. Later on, I was able to focus more on my own expertise.”*

*“Every appointment teached something new and strengthens my professional identity.”*

This progression suggests that structured repetition and increasing familiarity contributed to developing self-efficacy in clinical decision-making.

### 3.3. Enabling Conditions

Structured orientation was perceived as essential for safe and effective participation in patient care. Familiarization with clinical procedures and documentation practices reduced uncertainty and supported smoother clinical integration.

*“It was important for me to familiarize myself with the patient information system because I had no prior experience documenting in the system.”*

*“We were able to practice using the patient information system before the clinic sessions, and the study material was easy to follow, even on our own: In my opinion, the topics covered were therefore relevant from a practical standpoint.”*

Technical and licensing issues with patient information systems initially limited hands-on training time. Variability in patient complexity and preparation time also introduced uncertainty.

*“The orientation was a little confusing in terms of patient information system documentation, which I fully understand given that this is a pilot project.”*

*“The initial orientation was disrupted because we didn't receive our login credentials for the patient information system in time. I didn't have enough time to learn documenting properly, which caused uncertainty in the practice.”*

Despite these operational challenges, students framed them as part of the learning process within an authentic clinical environment.

“*In my opinion, the orientation went quite smoothly: I don’t think it’s even possible to provide very specific orientation for a course like this (and, on the other hand, facing a “challenge” right from the start isn’t a bad thing in terms of personal growth).*”

### 3.4. Skill Rehearsal and Role Integration

Independent pharmacist-led sessions provided a structured setting to rehearse medication reconciliation skills in a controlled environment. In contrast, joint interprofessional consultations required students to articulate their expertise within a team context, thereby promoting collaborative role integration.

“*I was able to apply the knowledge I had gained from my studies and work experience in a flexible manner. Got understanding my own role in teamwork: Trusting other professionals—I don’t have to do or know everything myself*.”

“*I learned about my working methods within an interprofessional team and how to develop my teamwork skills.”*

*“I learned a lot from the expertise of other professional groups. By listening to their conversations and observing how they interacted with patients, I learned new things myself.”*

This contrast suggests that independent sessions supported technical skill development, whereas interprofessional appointments fostered professional role negotiation and collaborative competence.

## 4. Discussion

This study provides insight into how pharmacy students can be meaningfully integrated into student-led interprofessional primary care clinics when their participation is supported by clear role expectations, supervised clinical activity, and structured reflection. Rather than positioning pharmacy involvement as an isolated medication-focused contribution, the course enabled students to move between independent pharmacist-led tasks and joint interprofessional consultations, making their expertise visible within team-based care. The findings suggest that authentic participation in a clinical learning community can support professional identity formation by helping students understand not only what pharmacists know, but how they contribute to collaborative patient care.

### 4.1. Professional Identity and Role Integration

The primary learning aims identified in this educational model; such as role clarification, professional identity formation, and the development of effective educational delivery; are strongly supported by the broader literature on interprofessional student-led clinics. Prior research consistently demonstrates that interprofessional learning environments help students articulate their own professional roles, understand the roles of others, and negotiate boundaries within collaborative practice.^2-5,10,20,21^ These processes are repeatedly highlighted as central mechanisms through which interprofessional education fosters professional identity development.

Systematic reviews of student-led and interprofessional clinics further show that participation in authentic, team-based service settings enhances students’ confidence, autonomy, and sense of professional belonging, while simultaneously challenging them to navigate ambiguity and negotiate responsibilities across disciplinary lines.^3-5,9^ Such findings align closely with the experiences reported in this study, where students described gaining clarity about their professional contributions and developing a more coherent sense of themselves as emerging practitioners.

Moreover, recent qualitative work underscores that identity formation in interprofessional contexts is not merely an individual cognitive process but is shaped by relational interactions, shared decision-making, and exposure to real patient care. This resonates with our findings, in which students emphasized the value of collaborative encounters and the opportunity to enact their roles in a supervised yet authentic clinical environment. Taken together, the evidence suggests that student-led interprofessional clinics provide a particularly fertile context for the development of professional identity, reinforcing the relevance and potential of this educational mode.

### 4.2. Structural Features Supporting Interprofessional Learning

Structured end-of-day reflection sessions were critical in consolidating interprofessional learning. Prior research has emphasized that structured support, dedicated time and active facilitation are essential for meaningful IPE outcomes.^6,7,10^ Guided reflection and learning diaries further supported deeper understanding of professional roles and collaborative practice.^22^

The clinic’s design as a dedicated educational unit, along with its strong interprofessional culture, supported the seamless integration of pharmacy activities—an element identified in prior research as central to cultivating a strong clinical learning environment.^6^ Furthermore, the unit also provided a unique platform for IPE by enabling simultaneous collaboration among multiple professions. From a CoP perspective, the Osmo Clinic functioned as a legitimate participation environment in which students could observe, engage, and gradually assume responsibility.^18^ CoP frameworks have been widely associated with professional identity development in healthcare education.^8,23^

### 4.3. Learner Characteristics and Implementation Considerations

Selecting advanced fifth-year students proved beneficial to test a new course in a practical clinic setting requiring adaptability and tolerance for structural adjustments. Their readiness for practice and voluntary participation likely facilitated engagement. Prior research has emphasized the importance of learner readiness and workplace participation in successful experiential learning.^24^ If the course had been compulsory and for lower-year students, self-initiative and adaptability might not have been at the same level.

### 4.4. Implications for Pharmacy Education

Pharmacy education in Finland is still developing its structures for practice-based learning and interprofessional training, particularly when compared with countries where experiential education is more firmly embedded in the curriculum.^11-14^ As noted earlier, Finnish pharmacy students have limited opportunities for patient-care-oriented clinical training during their studies,^13-15^and structured interprofessional outpatient models remain rare. Theis study demonstrates that student-led interprofessional clinics can offer pharmacy students meaningful, authentic learning experiences that support role clarity, collaborative competence, and professional identity formation. Expanding similar practice-based IPE opportunities within Finnish pharmacy education could help bridge the current gap and better prepare students for increasingly collaborative healthcare environments.

Introducing similar models earlier in pharmacy education may further enhance professional development, as early experiential learning has been associated with increased self-efficacy, practice readiness, and professional confidence.^10,25-27^ However, implementation with less advanced students would require expanded orientation and structured supervision. Scaling and standardizing the model would enable more rigorous evaluation of interprofessional learning outcomes, including potential effects on clinical reasoning, collaborative competence, and patient care processes. Scaling up would also mean a need for more resources. Recent evidence shows that student-led clinics produce benefits that reach far beyond the university, yet most evaluations capture only a narrow part of their impact.^28^ Existing studies highlight educational, societal, and health-system gains, while the operational costs fall mainly on universities and other educational institutions. Because evaluations rarely consider both costs and benefits, decisions about developing or sustaining student-driven outpatient models risk being based on partial information. For educational development, this underscores the need for multi-perspective evaluation approaches that account for learning outcomes, patient access, community impact, and health-system value alongside institutional costs.

The course demonstrated a promising educational model for integrating more clinically and patient-oriented IPE activities into the Finnish pharmacy curriculum, where such opportunities have traditionally been limited. Students perceived the educational model as both relevant and forward-looking within the Finnish healthcare context.

“*Overall, I am glad that I participated in this course. Although the course was still in its pilot phase, that did not bother me. It was interesting to learn about hospitals and health centers and to see how pharmacy professionals could be utilized more in the future.”*

### 4.5. Limitations

This study has several limitations. The small number of voluntary participants and single-site design limit transferability. As data were collected only from pharmacy students, the perspectives of patients, other professional student groups, and supervising clinicians were not captured. The reliance on self-reported reflections and feedback may also introduce social desirability bias. Another limitation of this study is that patient outcomes or user experiences have not been examined. Previous studies have found that both interdisciplinary approaches and student-led clinical placements have positive outcomes and user experiences.^1,3,29^ Finally, the six-week duration limits conclusions about longer-term effects on professional identity, collaborative competence, or clinical practice. Future studies should include larger, multiprofessional cohorts and mixed-method evaluation of learning outcomes and patient care processes.

## 5. Conclusion

Structured integration of pharmacy students into authentic student-led interprofessional clinics can support professional identity formation, collaborative competence, and clinical readiness. While empirically grounded in pharmacy education, the underlying principles of structured role development, supervised collaboration, and guided reflection may be relevant for experiential learning across health professions education.

## Supplemental Material

Supplemental material - Developing Professional Identity Through a Student-Led Interprofessional Clinic: A Qualitative Study in Experiential Pharmacy Education Supplemental material for Developing Professional Identity Through a Student-Led Interprofessional Clinic: A Qualitative Study in Experiential Pharmacy Education by Ville Valkonen in Journal of Medical Education and Curricular Development.

Supplemental material - Developing Professional Identity Through a Student-Led Interprofessional Clinic: A Qualitative Study in Experiential Pharmacy Education Supplemental material for Developing Professional Identity Through a Student-Led Interprofessional Clinic: A Qualitative Study in Experiential Pharmacy Education by Ville Valkonen in Journal of Medical Education and Curricular Development.

## Data Availability

Upon reasonable request.*
