# Supplementary material for: Developing Professional Identity Through a Student-Led Interprofessional Clinic: A Qualitative Study in Experiential Pharmacy Education
Source: J Med Educ Curric Dev. 2026 Jun 3;13:23821205261458512. doi: 10.1177/23821205261458512 (PMC13234492; doi:10.1177/23821205261458512)
Supplement: Supplemental material - Developing Professional Identity Through a Student-Led Interprofessional Clinic: A Qualitative Study in Experiential Pharmacy Education [file sj-pdf-2-mde-10.1177_23821205261458512.pdf]

UNIVERSITY OF EASTERN FINLAND
RESEARCH ETHICS COMMITTEE

Description No 52/2025

# To whom it may concern,

Clinical Lecturer Ville Valkonen (of study "Pharmacy Thrives in a Student-Led Interprofessional Clinic: Insights from Finland") has asked University of Eastern Finland (UEF) Research Ethics Committee to provide a description of the ethical review system for research in Finland.

In Finland, research with human participants must comply with the guidelines of the Finnish National Board on Research Integrity TENK[1] *The ethical principles of research with human participants and ethical review in the human sciences in Finland. Finnish National Board on Research Integrity TENK guidelines 2019* (pdf).

UEF has undertaken to comply with TENK's guidelines. The guidelines do not cover medical research as defined by law (Medical Research Act 488/1999) or other research designs where ethical review is a separate obligation laid down by law.

According to the guidelines, research is to be conducted in such a way that the dignity and autonomy of human research participants is respected and the research does not cause significant risks, damage or harm to research participants, communities or other subjects of research.

Ethical review is to be carried out prior to gathering data, if the research contains one or more of the following factors:

1. Participation in the research deviates from the principle of informed consent. Participation is not, for example, voluntary, or the subject is not given sufficient or correct information about the research.
2. The research involves intervening in the physical integrity of research participants.
3. The focus of the research is on minors under the age of fifteen, without separate consent from a parent or carer, or without informing a parent or carer in a way that would enable them to prevent the child's participation in the research.

---

[1] The Finnish National Board on Research Integrity TENK is an expert body appointed by the Ministry of Education and Culture and tasked with promoting research integrity and preventing research misconduct in Finland. Further information about the ethical review system in Finland is available at www.tenk.fi.

4. Research that exposes participants to exceptionally strong stimuli.
5. Research that involves a risk of causing mental harm that exceeds the limits of normal daily life to the research participants or their family members or others closest to them.
6. Conducting the research could involve a threat to the safety of participants or researchers or their family members or others closest to them.

**If none of the above factors is met, ethical review is not required.**

In Finland, neither legislation nor TENK's guidelines require ethical review by an ethics committee for research based purely on public and published data, registry and documentary data or archive data.

On 3rd December 2025

Mia Kilpeläinen, Chair

Mika Saikkonen, Secretary
